# Identifying Bilingual Children at Risk for Language Impairment: The Implication of Children’s Response Speed in Narrative Contexts

**DOI:** 10.3390/children8020062

**Published:** 2021-01-20

**Authors:** Pui Fong Kan, Anna Miller, Sierra Still

**Affiliations:** Department of Speech, Language, and Hearing Sciences, University of Colorado Boulder, Boulder, CO 80309, USA; anmi8839@colorado.edu (A.M.); Sierra.Still@colorado.edu (S.S.)

**Keywords:** processing speed, bilingual, impairment, screening

## Abstract

The purpose of the study was to examine whether monolingual adults can identify the bilingual children with LI on the basis of children’s response speed to the examiner. Participants were 37 monolingual English-speaking young adults. Stimuli were 48 audio clips from six sequential bilingual children (48 months) who were predominately exposed to Cantonese (L1) at home from birth and started to learn English (L2) in preschool settings. The audio clips for each child were selected from an interactive story-retell task in both Cantonese and English. Three of the children were typically developing, and three were identified as having a language impairment. The monolingual adult participants were asked to judge children’s response times for each clip. Interrater reliability was high (Kalpha = 0.82 for L1; Kalpha = 0.75 for L2). Logistic regression and receiver operating characteristic curves were used to examine the diagnostic accuracy of the task. Results showed that monolingual participants were able to identify bilingual children with LI based on children’s response speed. Sensitivity and specificity were higher in Cantonese conditions compared to English conditions. The results added to the literature that children’s response speed can potentially be used, along with other measures, to identify bilingual children who are at risk for language impairment.

## 1. Introduction

Developmental language disorder (DLD) [1] affects approximately 7–11% of children [2,3]. Children with DLD exhibit significant language deficits that cannot be attributed to sensory, motor, neurological, or socio-emotional impairments [1,3,4]. Clinically, one pressing problem is that young children who learn a minority language (L1) at home from birth and start to learn a community language (L2) in school settings are at particular risk for misdiagnosis with DLD [5,6,7,8,9]. There are three factors related to the over-identification and under-identification: (1) the fluctuation of typically developing (TD) bilingual children’s language skills as a function of L1 and L2 input and use [10,11,12], (2) the lack of valid language assessment tools and norms for bilingual children [13,14], and (3) the shortage of bilingual or bicultural speech-language pathologists who are trained to assess bilingual children [15,16].

In the past decades, many alternative assessment approaches (e.g., processing tasks) have been proposed to screen bilingual children who might be at risk for language impairment [17,18,19,20,21,22]. One approach that involves examining bilingual children’s processing speed has gained traction as the indicator for language impairment [20,21,22,23,24]. According to the processing-based accounts, the slow processing speed in children with DLD might be related to their limited ability to process linguistic information [25,26] and attention deficits [21,27]. Convergent evidence indicates that monolingual children with DLD demonstrate slower processing speed than TD monolingual children on linguistic and nonlinguistic tasks [23,25,28,29,30]. For example, children with DLD are slower to name pictures [31], judge whether a sentence is grammatically correct [24,32], recall visuospatial information [28], distinguish nonlinguistic tones [21,22,26], and rotate shapes mentally [23,30]. Park and colleagues [33] examined whether linguistic and nonlinguistic processing speed measures can be used as clinical markers for monolingual children with DLD. The binary logistic regression results showed that a combination of linguistic and nonlinguistic processing speed tasks moderately predict monolingual children’s DLD status. However, slow processing speed appears to be more predictive of the presence of DLD, but not the absence of DLD.

Bilingual children’s language experience is an important factor in the investigation of processing speed. Some studies found that bilingual experience could enhance TD bilingual children’s executive function, resulting in faster processing speed in certain nonlinguistic processing tasks that involve conflict resolution (e.g., card sort task) [34,35]. However, some studies do not find such an advantage in other processing tasks (e.g., visually detecting colors) [22,24]. These findings suggest that the variability in bilingual children’s response time could be associated with their bilingual experience, and the type of tasks could affect the diagnostic accuracy of processing speed. Ebert and Pham (2019) compared the processing speed of Spanish-English school-aged bilingual children with DLD (*n* = 92; 6; 0–10; 11) and aged-matched TD bilingual children (*n* = 109) using a nonlinguistic task, called visual detection. The task required the child to press a button that corresponded to the red/blue circle on the computer screen. They found that bilingual children with DLD were slower than their TD peers across all age groups. However, the sensitivity (ranging from 0.41 to 1) and specificity (ranging from 0.27 to 0.9) varied across age groups. Another consideration is the implementation of processing speed tasks for 3- to 5-year-old preschool children. Many processing tasks used in previous studies are designed for school-aged children and require the press of a button or strike of a key on a computer [21,22,23,27]. These tasks, which require attention control, motor, and perceptual skills to encode auditory and/or visual stimulus, might be difficult for young preschool-aged children [22,23,36]. The implementation of such processing speed tasks for young bilingual preschool children could lead to larger variability, which could negatively affect its diagnostic accuracy.

In this study, we present an alternative method for identifying young bilingual preschool children who are slower than their peers. Specifically, we examined the feasibility of using a judgment task by adults to identify the slow bilingual children who might be at risk for language impairment. Parental and teachers’ concerns, or ratings have been an important early indicator of developmental issues in clinical settings [37,38,39,40,41,42]. Many pre-screening and screening tools for bilingual children involve parents or teachers rating the amount of L1 and L2 input [10,38] and/or rating bilingual children’s language skills [43]. To our best knowledge, no previous studies have examined whether the rating of children’s response speed could be used as a tool for identifying at-risk bilingual preschool children who are at risk for language impairment in the screening process.

Previous research has utilized auditory-perceptual judgment tasks to examine speech characteristics such as respiration, voice quality, intelligibility, and fluency in individuals with speech disorders [44,45,46,47,48,49,50]. In this study, we explored whether judging children’s response speed to adults’ prompts in narrative contexts could be used to identify slow bilingual children who might be at risk for language impairment. Methodologically, two aspects should be noted in this investigation. First, the stimuli were extracted from the audio clips of three typically developing bilingual children who speak Cantonese as L1 and English as L2 and three Cantonese-English bilingual children who have been clinically identified as having language impairment. The interactive story-retelling task was implemented in two sessions for each child: one in Cantonese (children’s L1) and one in English (L2). Second, the response-speed judgment task is done by monolingual English-speaking adults. The use of monolingual adults was motivated by the shortage of bilingual clinicians in the U.S. The primary objective of this study was to examine whether monolingual adult speakers identify bilingual children with LI on the basis of their response speed to the examiner. The results would contribute to our understanding of the identification of at-risk bilingual children by monolingual clinicians. We specifically asked the following questions:

1. What is the interrater reliability of the response speed ratings?
2. What are the classification accuracies of the response speed ratings at the audio clip level? Are there any differences between L1 and L2 audio clips?
3. How well do the response speed ratings differentiate bilingual children with LI from TD bilingual children?

## 2. Materials and Methods

This project has been approved by the Institutional Review Board of the University of Colorado Boulder on 9 November 2018 (Protocol #: 18-0277).

### 2.1. Participants

Participants were 37 monolingual English-speaking adults (28 females and 9 males) between 18 and 41 years old (Mean age = 23.35; SD = 5). They were recruited from the Department of Speech, Language, and Hearing Sciences (SLHS) at the University of Colorado, U.S. To qualify for this study, the individuals must meet the following criteria: (1) primarily use English in his/her daily lives, (2) have no knowledge of Cantonese; (3) must have completed at least two courses in SLHS. The participants reported that they had between 15 and 20 years of formal education, from first grade to their current educational year (Mean = 16.08 years; SD = 1.38). Of the 37 participants, 27 were undergraduate students, 8 were in the post-baccalaureate or master’s program, and 2 were in the doctoral program. Most of the participants (*n* = 27) were White; 3 were African American; 2 were Asian American, and 5 were mixed race. None of the participants had exposure to Cantonese. None of them reported that they had language, hearing, or vision problems.

### 2.2. Response-Speed Judgement Task

Stimuli of the Response-Speed Judgement Task were 48 short audio-clips (Mean = 23.7 seconds; SD = 9.83) of the adult-child interactions of 6 children (4 clips per child × 2 languages) during a story-retell task (see Table 1). These samples were selected from 248 audio recordings of a larger study led by the first author. All children had audio recordings in L1. The third author used *Praat* [51] to identify the examiner–child interactions in the audio recordings of the story-retell tasks in L1 and L2. The selection criteria of the clips included at least three continuous exchanges between the examiner and the child. Children who only had recordings in L1 or L2 were excluded. The children with LI and TD children were age-matched. Because of the variation of the adult–child interactions, the clips varied in times. The identity of the selected clips was blind to the rest of the research team; only the third author, who was not involved in data collection, had the key to all the selected clips. The 48 selected audio clips, including those from TD and LI groups, were randomly combined into two large audio files: one in Cantonese (24 clips) and one in English (24 clips). There was a five-second interval of silence between each clip.

The six children were exposed to Cantonese (L1) at home from birth and started to learn English (L2) when they started preschool. Three of the six children were typically developing (TD) children (2 females, 1 male). The other three children (two females, one male) were clinically identified as having language impairment (LI) and had an individual educational program (IEP). The clinical diagnoses were based on clinicians’ interpretation of children’s language performance on criterion-reference tasks, parents’ concerns, and teachers’ reports, and clinical observations. All children had a standard score of 80 or above on the brief IQ screening of the Leiter-R [52]. There were no significant differences between the two groups *F*(1, 4) = 1,29, *p* > 0.05. Since there are no valid measures and norms to make DLD diagnosis for Cantonese-English bilingual children [11], we use a broad term, language impairment (LI), to describe the children who received language intervention in this study. The story-retell task involved each child retelling a story, *Frog, Where Are You?* [53] after the examiner told him/her the story. The story retell task was administered in both Cantonese and English. The prompts by the examiner were open-ended and minimal, including phrases such as “tell me more”, “uh-huh”, “and then what happens?” to encourage the child to continue the story. The order of the language tested first was counterbalanced. Table 1 summarizes the information of the audio clips. To reduce biases, the measurements were done after the monolingual participants completed the response-speed judgment tasks.

Repeated measures analysis of variance (ANOVA) indicated that there was no significant effect of group (LI vs. TD clips) on clip length, *F*(1, 44) = 1.37, *p* > 0.05, or on turns *F*(1, 44) = 1.2, *p* > 0.05. There was no effect of language (Cantonese vs. English) on clip length, *F*(1, 44) = 1.96, *p* > 0.05, or on turns *F*(1, 44) = 0.61, *p* > 0.05. The findings suggest that the clips were comparable across the two groups and across languages. In terms of the responses by examiners, the repeated measures ANOVA results also showed that there was no significant group effect (LI vs. TD clips) on examiner syllables per second, *F*(1, 44) = 3, *p* > 0.05, or on examiner response to child interval *F*(1, 44) = 0.003, *p* > 0.05. There was no significant language effect on examiner syllables per second, *F*(1, 44) = 2.28, *p* > 0.05, or on examiner response-to-child interval *F*(1, 44) = 0.87, *p* > 0.05. The findings suggest that the prompts by the examiner and the amount of time the examiner took to respond to the child were comparable across the two groups and across languages. There was a significant group effect (LI vs. TD clips) on children’s syllables per second, *F*(1, 44) = 1.77, *p* < 0.05, suggesting there were fewer syllables per second in the clips of children with LI than those of TD children. There was a significant language effect on children’s syllables per second, *F*(1, 44) = 9.38, *p* < 0.05, suggesting children had more syllables per second in Cantonese than in English. The results are consistent with the teachers’ report that children had stronger Cantonese skills (L1) than English at the time of testing. Repeated measures ANOVA results showed that there was a significant group effect on response-to-examiner interval, *F*(1, 44) = 37.85, *p* < 0.001, suggesting children from the LI group took longer to respond to the examiner than their TD peers. There was no language effect, *F*(1, 44) = 0.44, *p* > 0.05 or group × language interaction on response-to-examiner intervals, *F*(1, 44) = 1.37, *p* > 0.05.

To illustrate the variability of individual children’s response-to-examiner intervals (in seconds), we summarize the means and standard deviations of the response-to-examiner intervals of each child in Table 2. There are four clips for each child for each language; and there were six to eight turns within each clip.

### 2.3. Procedures

Each participant was tested separately in a quiet room in the laboratory. It took the participant between 25 and 35 min to complete the practice trials and rate the audio clips in the testing phase. Practice trials were administered before testing to ensure that the participants understood the testing procedure. The practice trials involved four audio clips, which were different from those used for the response-speed judgment task. Two of the practice clips contained interactions of a child who had slow response speed, while the other two practice clips were interactions of a child who had normal response speed. The examiner read the following script to each participant: “You will listen to a series of audio-clips, where you will hear an adult and a child’s voice. Please rate the speed that you believe it takes the child to respond to the examiner. The scale ranges from 1 to 4. “1” is a very slow response time, “2” is a slow response, “3” is a slightly slow response, and “4” is a normal response time.” After the presentation of each clip, the participants were instructed to mark the child’s response speed on a 4-point scale. To advance the response-speed judgment task, the participants had to respond correctly to all four practice items and verbally indicate that they understood the procedures. All participants reached the criteria.

During the response-speed judgment task, the 48 target audio clips were presented to each adult participant. The participants were not told that some clips were from children with language impairment, and some were from TD children. The order of the Cantonese and English clips was counterbalanced. Nineteen participants were presented to the 24 Cantonese clips before the 24 English clips; 18 participants were presented to the 24 English clips prior to the 24 Cantonese clips. Before each clip was presented, a number was shown on the computer screen in front of the participant to confirm the clip number, which corresponded with the rating form. After the examination of interrater reliability (see Section 3.1), the ratings of 1 and 2 were coded as “slow speed” and 3 and 4 as “normal speed” for analysis.

## 3. Results

### 3.1. Interrater Agreement

Krippendorff’s α was computed to examine the reliability across the 37 raters for items in each language. The Krippendorff’s α was developed for more than two raters and various data types, including ordinal data [54]. In this analysis, each rater’s ratings, ranging from 1 to 4, were examined. The 95% confidence intervals (CIs) were calculated by bootstrapping (*n* = 10,000). As shown in Table 3, Kalpha was 0.82 (95% CI = 0.81, 0.82) for the Cantonese items, suggesting high interrater agreement about the response speed of the children in the clips in Cantonese, whereas Kalpha was 0.75 (95% CI = 0.75, 0.76) for the English items, suggesting moderate interrater agreement about the response speed of the children in the clips in English.

### 3.2. Slow Speed Ratings and Audio Clips from Children with LI

The ratings of 1 and 2 were coded as “slow speed” and 3 and 4 as “normal speed” for analysis. The distribution of slow response speed ratings is summarized in Table 4. Overall, 95% of the clips from the children with LI in the Cantonese condition were identified as slow speed, whereas 77% of the clips from the children with LI in the English condition were identified as slow speed.

**Figure 1:**
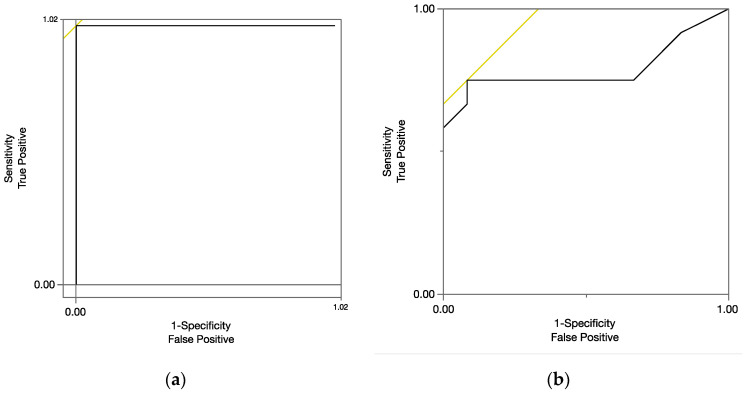
Receiver operating characteristic (ROC) curve for slow rating for all audio clips, (**a**) Cantonese; (**b**) English.

Logistic regression analyses showed that the clips from children with LI in the Cantonese conditions were likely to be rated as “slow speed,” χ^2^ (1) = 33.27, *p* < 0.001; and children with LI in the English conditions were likely to be rated as “slow speed,” χ^2^ (1) = 9.31, *p* < 0.01. The ratings were assessed as a metric for determining the clip categories (i.e., LI or TD). Receiver operating characteristic (ROC) curves are plotted in Figure 1 (1a for the Cantonese and 1b English) and the areas under the ROC Curve (AUC) were calculated. For the Cantonese conditions (24 clips), the sensitivity was 1, and the specificity was 1, with AUC = 1 (see Table 5). The results indicated that the ratings were excellent at separating the audio clips of children with LI from those of TD children (see Figure 1a). For the English conditions (24 clips), the sensitivity was 0.7 and the specificity was 0.92, with AUC = 0.79 (see Table 5). The results indicated that the ratings were good at separating the clips of children with LI from those of TD children (see Figure 1b).

### 3.3. Identification of Children with LI Using Response Speed Ratings

**Figure 2:**
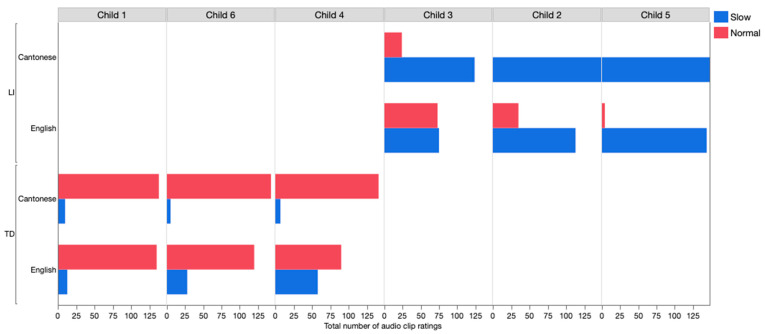
Total number of audio clip ratings (slow vs. normal) for each child. TD = Typically-developing and LI = Language impairment

Figure 2 displays the total number of audio clip ratings for each child (37 participants × 4 clips for each language). For the Cantonese conditions with four clips nested within each child, the ratings (slow vs. normal) predict LI, χ^2^ (4) = 33.27, *p* < 0.001. The findings suggest that the ratings of the Cantonese samples are excellent in differentiating the children with LI from TD children. For the English conditions with four clips nested within each child, the ratings (slow vs. normal) predict LI, χ^2^ (4) = 19.14, *p* < 0.01. The findings suggest that the ratings of the English samples are likely to differentiate the children with LI from TD children. However, there are some individual differences across children. As shown in Figure 2, the ratings for Child 3 (with LI) and Child 4 (TD) for the English condition, were at chance or almost at chance, respectively, although the ratings for these children’s Cantonese clips identify Child 3 as LI and Child 4 as TD.

## 4. Discussion

The present study was built from the literature about the slow processing speed in children with DLD [21,25,26,33]. The study was designed to examine young bilingual children who cannot complete the processing tasks. Rather than directly testing young children, this study examined whether monolingual adults’ judgment of children’s response speed in a narrative context could be used to identify bilingual children with LI (mean age = 48 months). Thirty-seven normal monolingual English-speaking adults (28 females and 9 males), who were enrolled in the SLHS Department, completed a response-speed judgment task. The task stimuli were 48 audio clips of 6 bilingual preschool children who began learning Cantonese (L1) from birth and English (L2) in a preschool setting. Three of these children had been identified as having language impairment, and three were typically developing bilingual preschoolers. The audio clips for each child were selected from an interactive sample between an examiner and a child, where he/she was asked to retell a story called, *Frog, Where Are You?.* Several key findings emerged from the analyses of this study. First, reliability is particularly notable across the 37 raters for both Cantonese and English audio clips. Second, both sensitivity and specificity were high for audio clips (Figure 1). Importantly, although the raters do not know Cantonese, they were able to identify the audio clips of the children who were slow in responding to the examiners. Their ratings were more consistent when rating clips in Cantonese than in English. Third, variability was noted across raters in the English clips nested within individual children. The English samples of two children, in particular, were rated at chance or near chance level.

One important finding in this investigation is the high agreement among raters. The participants were asked to rate the child’s responses to the examiner on a 4-point scale. It is important to note that because the audio samples were taken from interactive narrative samples, there was great variability in the response speed among the audio clips from each group of children (see Table 2). The 37 monolingual English-speaking participants received limited training about response speed before the task began. Yet, high interrater agreements about the response speed of the children in the clips were found for the Cantonese items (Kalpha = 0.82; 95% CI = 0.81, 0.82) and for the English items (Kalpha = 0.75; 95% CI = 0.75, 0.76). This finding indicates monolingual clinicians or teachers could rate the response speed with a high agreement level. Future research is needed to replicate the high interrater agreement in the response-speed judgment task using stimuli from bilingual children who learn other languages as a home language.

In this study, the primary question is whether the response speed rating (slow vs. normal speed) by monolingual adults predict the audio clip type (LI vs. TD). The 48 audio clips were from 6 Cantonese-English bilingual children: 3 with LI and 3 TD children. Consistent with prior findings [22,24,26], children with LI, as a group, had significantly slower response speed than TD children in both Cantonese and English conditions (see Table 2). Although the monolingual English-speaking participants did not speak Cantonese (L1), they could still identify children who were slower to respond in Cantonese. For the Cantonese clips, the monolingual adults’ ratings appear to have excellent sensitivity and excellent specificity (100% and 100%, respectively; Figure 1a). For the English clips, the ratings by the monolingual adults appear to have good sensitivity and excellent specificity (75% and 92%, respectively; Figure 1b). There are three possible explanations for the difference between the Cantonese and English conditions. The first explanation is related to the monolingual participants’ knowledge of English. The participants are monolingual English-speaking and have no knowledge of Cantonese. When making a judgment on the English clips, they could be distracted by the linguistic contexts. Although the participants were instructed to use response time in making their judgements solely, other linguistic cues (e.g., prosody, vocabulary, grammar, or intonation) in the audio clips could have implicitly affected their ratings. In contrast, because the participants did not have any exposure to Cantonese, they were likely to focus solely on children’s response speed. The second explanation is that the audio clips were sampled from children who were at the beginning stage of learning L2. These children, as a group, had more L1 experience and stronger L1 skills at the time of testing. As a result, the variability cross children’s response-to-examiner in the L2 clips were high (*SD* = 4.13 for the TD children) compare to the L1 clips (*SD* = 0.069 for the TD children). It is likely the variability for the clips in L2 contributes to the variability of ratings in L2. The third explanation might be related to the response speed difference between the Cantonese and English samples. For the Cantonese samples, the mean response speed of the TD children ranged from 0.64 to 0.75 s, but the response speed of the children with LI was between 1.48 to 5.5 s. For the English samples, the response speed of the TD children was from 0.88 to 1.66 s, but the mean response speed of the children with LI was from 1.96 to 2.25 s. The larger LI–TD contrast in the Cantonese clips may have contributed to the high sensitivity and specificity. Future studies are needed to investigate the LI-TD contrast across L1 and L2 samples in diagnostic accuracy.

The present study examined the response speed ratings of the audio clips nested within each child (four clips × two languages). One limitation is that the stimuli were developed using a small number of children’s narrative samples (*n* = 6). Although we had a larger database of language samples from Cantonese-English children with LI, many children with LI did not meet the selection criteria (e.g., three exchanges between the examiner and child for both Cantonese and English). Some children did not have three exchanges, while some children’s language samples in Cantonese met the selection criteria, but their English samples did not. Future work should use less restrictive criteria to include samples from more children to validate the response-speed judgment task. A second limitation is that the clips were selected from children’s narrative samples. The response speed to the examiner varies within each child. For the Cantonese samples, the overall ratings appear to differentiate children with LI from TD children accurately (Figure 2). However, the ratings for the English samples appear to be less accurate. In particular, the ratings for Child 3 (with LI) and Child 4 (TD) were at chance or almost at the chance, respectively. As noted in Table 2, Child 3, although had a diagnosis of LI, was faster than the other two children in the LI group in the English conditions (Child 2 and Child 5). In contrast, Child 4 did not appear to be significantly faster or slower than the other two TD children. Future investigation with larger samples is needed in order to examine the response speed threshold’s effect on listeners’ judgment.

In the search for screening tools across a wide variety of languages, this study explores the methodology that allows clinicians to identify at-risk children on the basis of their response speed. The findings in this study provide some preliminary evidence for including a response-speed judgment task as a screening tool for monolingual English-speaking speech-language pathologists who work with bilingual preschool children. Future work needs to be done to examine how judging children’s response speed is incorporated in classroom observation and parent or teacher reports.

## Figures and Tables

**Table 1:** Audio-clips information: LI and typically developing (TD) children by language.

	LI (12 Clips)		TD (12 Clips)	
	Cantonese	English	Cantonese	English
Clip length (in seconds)	19.92 (1.26)	21.42 (7.51)	19.83 (5.54)	26.50 (11.07)
Turns	6.00 (4.16)	6.75 (4.41)	7.08 (3)	8.00 (2.95)
Examiner syllables per second	4.77 (0.73)	4.07 (1.26)	5.02 (0.43)	5.17 (1.12)
Examiner response-to-child interval (in seconds)	0.30 (0.28)	0.30 (0.26)	0.24 (0.14)	0.36 (0.22)
Child syllables per second	2.04 (2.24)	2.05 (1.05)	4.85 (1.57)	2.14 (.86)
Child response-to-adult interval	3.24 (2.55)	2.25 (1.41)	0.67 (0.05)	1.2 (0.41)

Note. Child response-to-examiner interval = the interval between the end of the examiner’s prompt and the onset of the child’s first syllable; Examiner response-to-child interval = the interval between the end of the child’s utterance and the onset of the examiner’s first syllable.

**Table 2:** Mean and Standard Deviation of child response-to-examiner interval (in seconds).

	Group	Cantonese (L1)	English (L2)
Child 2	LI	2.75 (0.75)	2.56 (1.05)
Child 3	LI	1.48 (0.44)	1.95 (0.65)
Child 5	LI	5.5 (0.45)	2.25 (0.26)
Child 1	TD	0.75 (0.71)	1.66 (0.47)
Child 4	TD	0.64 (0.38)	1.06 (0.77)
Child 6	TD	0.63 (0.53)	0.88 (0.14)

Note: TD = Typically-developing; LI = Language impairment.

**Table 3:** Interrater agreement: Krippendorff’s α (kalpha) for ratings across 37 raters.

	Kalpha	95% CI	*p* (Kalpha < 0.60)
Cantonese (L1)	0.82	[0.81, 0.82]	<0.001
English (L2)	0.75	[0.75, 0.76]	<0.001

**Table 4:** Distribution of slow response speed ratings by audio clip category (LI vs. TD) by language.

	LI	TD
Cantonese (L1)	420 (95%)	22 (5%)
English (L2)	332 (77%)	99 (23%)

**Table 5:** Ratings associated with the best combination of sensitivity and specificity.

	X	Sensitivity	Specificity	False Positive	False Negative
Cantonese (L1)	13.00	1.0	1.0	0	0
English (L2)	34.00	0.75	0.92	0.08	0.25

## Data Availability

The data presented in this study are available on request from the corresponding author. The data are not publicly available due to original informed consent provisions.
